# Degenerative Cervical Myelopathy in a Patient With a Pre-existing Cervical Spinal Cord Stimulator: A Case Report

**DOI:** 10.7759/cureus.86204

**Published:** 2025-06-17

**Authors:** Chase Walton, Sarah Jenkins, Robert J Ferdon, James Lawrence, Robert Ravinsky

**Affiliations:** 1 Department of Orthopaedics and Physical Medicine, Medical University of South Carolina, Charleston, USA; 2 Department of Orthopaedic Surgery, University of South Carolina, Columbia, USA

**Keywords:** anterior cervical discectomy and fusion (acdf), degenerative cervical myelopathy, neuromodulation, spinal canal stenosis, spinal cord stimulator

## Abstract

Degenerative cervical myelopathy (DCM) is a progressive condition that can lead to significant neurologic disability if not promptly diagnosed and treated. The presence of a neuromodulation device in the spinal canal, such as a spinal cord stimulator (SCS), can potentially hinder the diagnosis and management of DCM. Here, we report a case of a 53-year-old female patient with a history of complex regional pain syndrome being managed with a cervical SCS, who presented with DCM and rapid neurological deterioration and bilateral lower extremity paralysis. The diagnostic workup was rendered more challenging due to contraindications for MRI related to the implanted SCS. CT myelogram demonstrated multilevel cervical stenosis with cord compression; the cervical leads were believed to contribute to the central stenosis. The patient underwent anterior cervical discectomy and fusion (ACDF) from C4 to C7, resulting in immediate improvement in her motor function and an uncomplicated postoperative course. At discharge, she had regained full strength in the lower extremities. This case highlights the unique diagnostic and management challenges of DCM in patients with pre-existing cervical neuromodulation devices. A high index of suspicion and timely use of alternative imaging modalities were essential for achieving favorable outcomes in this complex patient. Further research is needed to establish evidence-based recommendations for the management of DCM in the setting of neuromodulation devices.

## Introduction

Historically, degenerative cervical myelopathy (DCM) has been recognized for its insidious onset and gradual impairment of function, leading to significant disability if not promptly diagnosed and managed [1]. Advancements in diagnostic imaging and surgical techniques have significantly improved the management of DCM. However, management becomes particularly challenging in patients with pre-existing neuromodulation devices, such as spinal cord stimulators (SCS), which may hinder diagnostic imaging and contribute to spinal canal stenosis due to mass effect or fibrosis. SCS and other neuromodulation devices are commonly used to manage chronic neuropathic pain conditions, including complex regional pain syndrome, failed back surgery syndrome, and peripheral neuropathy [2-7]. While MRI compatibility with SCS devices has been explored in the literature [8,9], reports of the SCS itself contributing to DCM are exceedingly rare. Certain devices are conditionally MRI-compatible, depending on the strength of the magnet in the MRI machine, while others are incompatible [10]. Here, we present a unique case of a 53-year-old female patient whose cervical SCS both complicated the diagnosis and contributed to the pathogenesis of DCM, ultimately leading to rapid neurological deterioration. We aim to discuss the clinical presentation, diagnostic challenges, surgical management, and outcomes of this case, providing insights for clinicians managing similar complex scenarios.

## Case presentation

A 53-year-old female patient presented to the emergency department with progressive bilateral lower extremity weakness, acute-onset numbness in the lower extremities and perineal region, and urinary and fecal incontinence persisting for over two weeks. Relevant history included type II diabetes, hypothyroidism, complex regional pain syndrome managed with a cervical SCS (implanted in 2007), fibromyalgia, and cervical spinal stenosis. She denied tobacco, alcohol, or illicit drug use. On presentation, the patient required a wheelchair due to lower extremity paresis.

On physical examination, the patient demonstrated significant bilateral lower extremity weakness, rendering her unable to move her legs. There was a marked loss of sensation from the navel to the toes, with reported numbness. The clinical presentation of positive right-sided Hoffman's sign, 1-2 beats of clonus at the ankle bilaterally, saddle anesthesia, combined with bowel and bladder incontinence, was concerning for cord compression. No new surgical incisions or acute abnormalities related to her SCS implant were noted. Her Modified Japanese Orthopedic Association score was 9, indicating significant neurological impairment. The upper limb motor score was 5, the lower limb motor score was 1, the upper limb sensory score was 3, and the sphincter score was 0.

**Figure 1:**
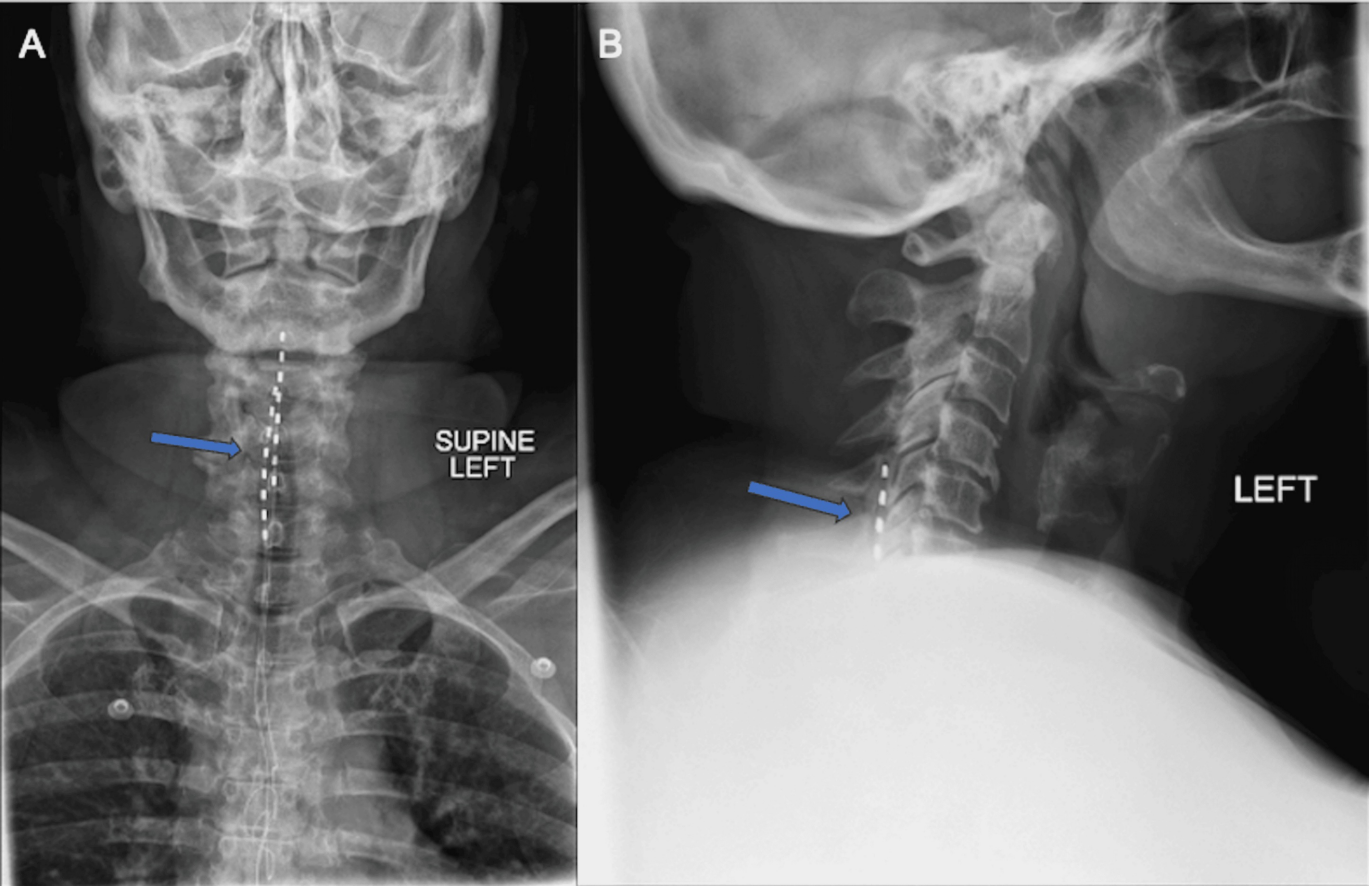
Preoperative anterior-posterior (AP) (A) and lateral (B) films of the spine demonstrating multilevel degenerative changes and spinal cord stimulator (SCS) leads in the cervical and thoracic spine. The blue arrow demonstrates the presence of the SCS leads in the dorsal aspect of the spinal canal in both AP and lateral views.

**Figure 2:**
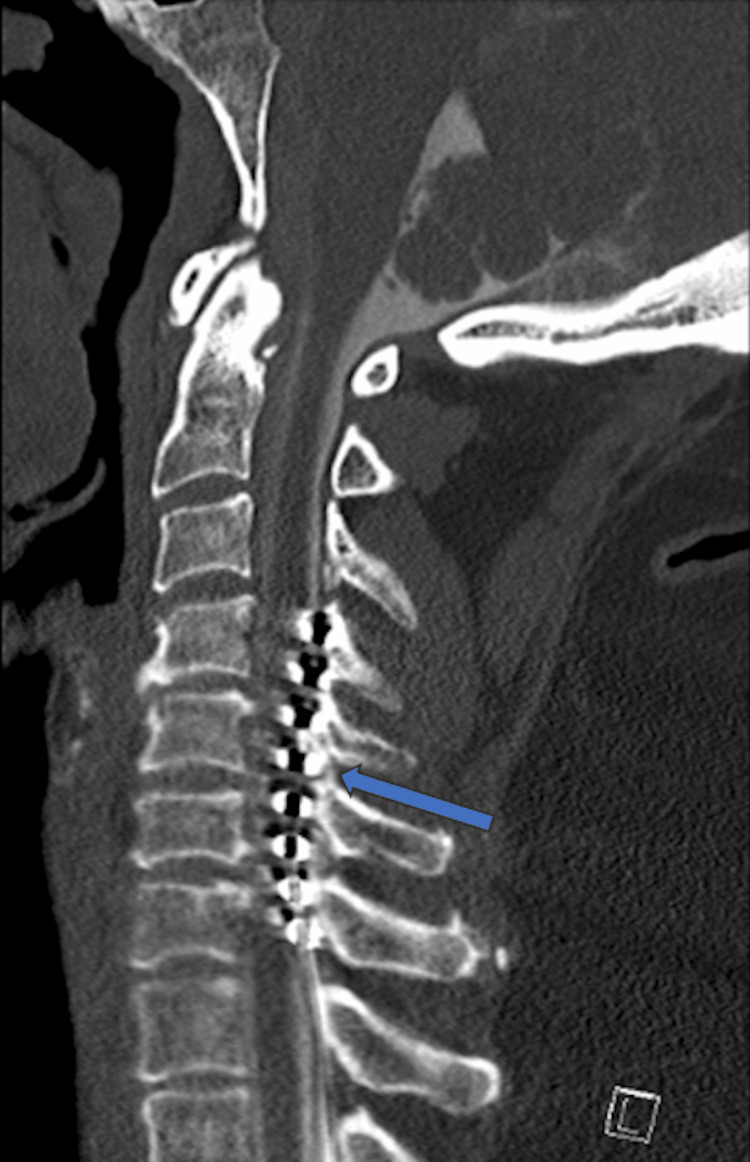
Preoperative sagittal CT scan of the cervical spine re-demonstrating spinal cord stimulator (SCS) leads in the cervical spine. The blue arrow denotes the presence of the SCS leads in the dorsal aspect of the spinal cord canal.

**Figure 3:**
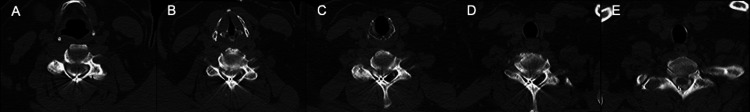
Preoperative axial CT images revealing high-grade central stenosis at C4-C7 with contribution to stenosis from spinal cord stimulator leads. Axial images include levels C3-C4 (A), C4-C5 (B), C5-C6 (C), C6-C7 (D), and C7-T1 (E).

Radiographic imaging demonstrated multilevel degenerative changes and the presence of SCS leads in the cervical and thoracic spine (Figure 1). The diagnostic workup was complicated by the patient's SCS being incompatible with 3.0 T MRI. A CT myelogram revealed multilevel cervical spondylosis and severe canal stenosis from C4 to C7, with the SCS leads causing a mass effect on the spinal cord (Figures 2, 3). Given the severity of symptoms and radiographic findings, an anterior cervical discectomy and fusion (ACDF) from C4 to C7 was recommended to preserve the integrity of the posterior spinal elements, which is particularly advantageous in the context of the implanted SCS.

**Figure 4:**
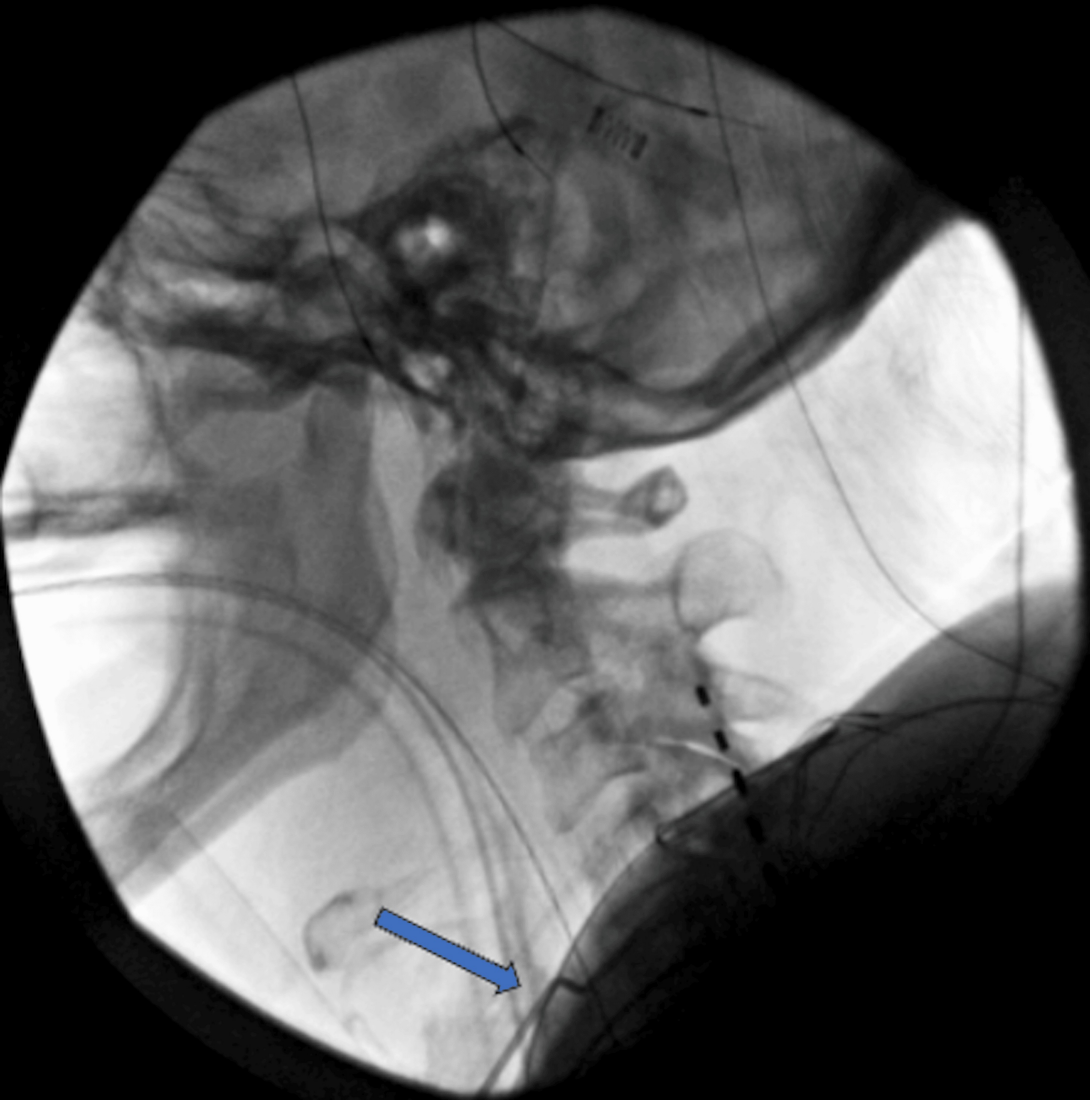
Intraoperative lateral fluoroscopic localizer image with a fiducial marker in the vertebral disc space. The blue arrow denotes the presence of a bent spinal needle as a fiducial marker in the C4-C5 disc space.

After achieving total intravenous anesthesia and obtaining baseline neurophysiological monitoring, the patient was positioned supinely on a Jackson flat top table with her arms tucked and shoulders secured with tape to facilitate lateral fluoroscopic imaging. A right-sided transverse incision was made, followed by a standard Smith-Robinson approach dissection through the anatomical layers using electrocautery and sharp dissection, accessing the anterior aspect of the cervical spine. After completing exposure, a fiducial marker was used to localize the vertebral disc spaces (Figure 4). Subtotal discectomies were performed at C4-5, C5-6, and C6-7, removing disc material and osteophytes, followed by decompression of the nerve roots and spinal cord. Appropriate-sized interbody cages filled with bone graft were implanted, and a stabilizing anterior plate was affixed. The surgery concluded with multilevel motor evoked potential checks to ensure neurological integrity, confirming no significant changes from baseline. The wound was closed in layers, ending with the application of a sterile dressing. The patient was transferred to postanesthesia care in stable condition. The procedure was completed without complications, and intraoperative neuromonitoring remained stable throughout.

**Figure 5:**
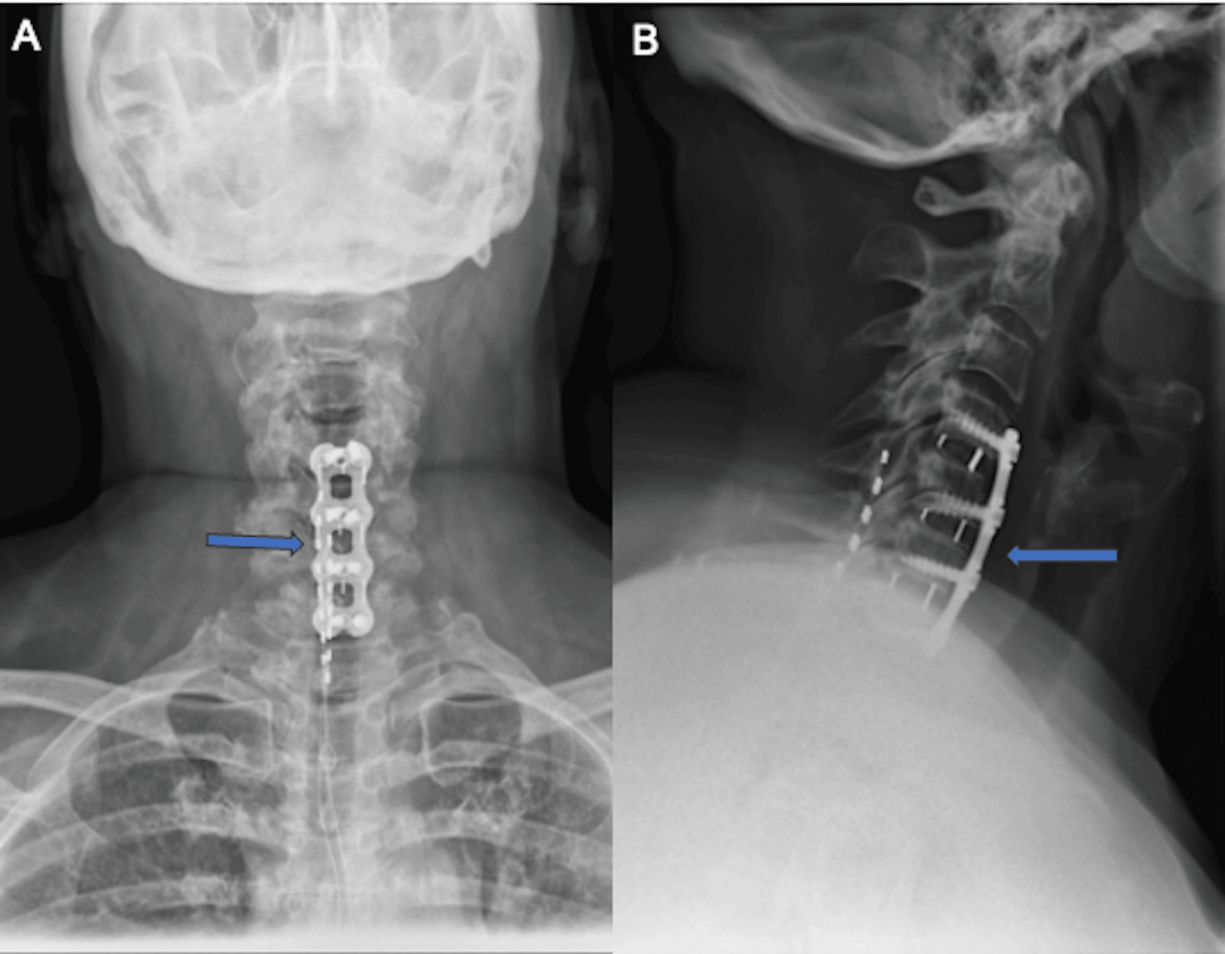
Postoperative follow-up anterior-posterior (AP) (A) and lateral (B) films of the cervicothoracic spine, confirming appropriate implant placement and alignment without complications. The blue arrow denotes the presence of a stabilizing anterior plate and interbody cages in both AP and lateral views.

Postoperatively, the patient demonstrated significant improvement in neurologic function. Within hours after surgery, she experienced significant improvement in her symptoms, with increased strength in her lower extremities at the immediate postoperative check. A cervical orthosis was applied, and physical therapy was initiated as part of her postoperative care regimen. At the three-week postoperative visit, the patient reported continued symptomatic improvement, with only mild residual numbness in her lower extremities. There were notable improvements in both lower extremity strength and sensory deficits. Radiographs confirmed appropriate implant placement and alignment without complications (Figure 5). By week 6, the patient showed continued progress. She had begun physical therapy, and her pain was well-controlled with medication. Neurological exams and follow-up radiographs confirmed early fusion without complications and marked improvement in outcome measures. At three months postoperatively, the patient reported a substantial reduction of her presenting symptoms, with marked improvement in lower extremity strength and decreased sensory deficits.

## Discussion

Cervical myelopathy is a progressive condition caused by spinal cord compression resulting from degenerative changes, such as disc bulging and osteophyte formation [11]. The pathophysiology includes both static factors, which result in a progressive decrease in space available for the cord, as well as dynamic factors from segmental motion [12]. Although symptom progression is usually gradual, rapid neurological decline can occur, as demonstrated in the present case [1]. In this patient, the presence of a cervical SCS (Abbott Proclaim model) with 60 cm long leads and 1.3-1.4 mm in diameter exacerbated the symptoms of cervical myelopathy by contributing to spinal canal stenosis.

Epidural fibrosis resulting in spinal cord compression is a rare, delayed complication that may occur five to 17 years post-implantation of cervical SCS leads. Etiologies may involve electrical stimulation, postoperative blood/serum, foreign body reaction, or low-grade infection [13]. Epidural fibrosis is more frequently observed with cervical leads than thoracic leads, due to greater cervical spine mobility and smaller spinal canal diameter. Symptoms include progressive myelopathy, paresthesia, gait ataxia, weakness, and increased muscle tone and reflexes, often accompanied by decreased stimulation effectiveness [14]. Epidural fibrosis can occur even with percutaneously placed leads and is not limited to laminectomy cases.

Diagnosing epidural fibrosis is challenging due to the presence of imaging artifacts from the SCS leads. In this case, the diagnosis of DCM was complicated by MRI contraindications, including limited compatibility and significant imaging artifacts. Although MRI following lead removal offers the best visualization, it is often impractical before intervention. Clinical assessment is also complicated by symptom overlap between DCM and conditions treated by SCS. Additionally, managing SCS device settings during imaging presents challenges. Studies have demonstrated that while MRI can be performed in patients with high-frequency SCS devices under specific protocols, the presence of the SCS devices still presents significant limitations and risks, which were pertinent in our patient's diagnostic process [8,10]. Thus, alternative imaging like CT myelography may help visualize cord compression despite less detail [15]. In this case, CT myelography revealed high-grade cervical central stenosis, and given the dramatic presentation and presence of SCS, surgical intervention was recommended.

ACDF is a well-established surgical treatment for DCM, which can effectively decompress the spinal cord and restore stability to the affected segments [16]. However, in patients with SCS, additional preoperative planning and intraoperative precautions are necessary to avoid device-related complications. In this case, the surgery was performed without altering the standard ACDF technique, but careful consideration was given to the presence of the SCS leads to prevent further spinal canal compression and ensure device integrity. The patient underwent a successful ACDF from C4 to C7, resulting in significant improvement in her preoperative symptoms and an uncomplicated postoperative course. These findings are consistent with the generally favorable outcomes reported for ACDF in the treatment of DCM.

While the surgical procedure itself was standard, the presence of the SCS added complexity to the management of the patient. The SCS leads not only contributed to spinal canal stenosis but also necessitated meticulous surgical planning to avoid damaging the device and ensure simultaneous thorough decompression of the spinal cord. In typical cases of epidural fibrosis due to SCS leads, surgical treatment involves the removal of both the stimulator lead and the fibrous mass, usually found surrounding the lead, compressing the spinal cord [17]. However, in our case, the SCS leads were not removed, as the primary issue was mechanical compression from the degenerative changes exacerbated by the leads. The SCS was providing relief for severe, debilitating chronic regional pain syndrome. As such, the patient expressed she would prefer to retain the SCS if possible. We felt that retaining the device while performing an anterior decompression was both technically feasible and afforded the least surgical morbidity. Furthermore, surgical removal of the SCS followed by MRI prior to rendering a decision about the management of her cervical stenosis was an impractical option that would lead to a delay in care, which in turn could have compromised the neurological outcome. This approach may differ from typical cases of epidural fibrosis, highlighting the unique considerations required when managing DCM in patients with pre-existing, well-functioning SCS devices. As the utilization of neuromodulation devices continues to increase, it is important for healthcare professionals across various disciplines to be aware of the potential implications of these devices in the setting of central cervical stenosis and DCM, as well as other spinal pathologies.

Our case underscores the importance of recognizing the physical presence of SCS leads as a potential contributing factor to spinal canal stenosis, highlighting the need for clinicians to remain vigilant in patients presenting with new or worsening neurological symptoms. Furthermore, it emphasizes the necessity of integrating existing literature on MRI compatibility and the unique challenges of SCS devices into clinical decision-making processes.

Future research efforts should focus on developing evidence-based guidelines for the diagnosis and treatment of DCM in the presence of neuromodulation devices. Key areas of focus should include device compatibility with imaging modalities and surgical techniques, as well as strategies for minimizing device-related complications. Future research should also explore potential modifications to lead design and materials to minimize fibrosis. Additionally, studies should investigate pharmaceutical interventions to prevent or reduce epidural fibrosis in SCS patients [14]. As the field of neuromodulation continues to evolve, ongoing research will be essential for refining care pathways and improving outcomes for patients with DCM and other structural pathologies who are also concurrently being treated with neuromodulation for centralized neuropathic pathologies.

## Conclusions

This case report highlights the complexities of treating cervical myelopathy in patients with pre-existing cervical neuromodulation devices and presents a reasonable management strategy despite diagnostic challenges. Given the increasing utilization of SCS, we feel that this scenario may become increasingly common with time. Understanding the clinical features of cervical myelopathy, even in the context of neuromodulation, is crucial for developing refined guidelines that ensure optimal outcomes for this unique patient population. Through detailed clinical and radiographic evaluation, coupled with a strategic surgical approach, immediate and significant improvements in the patient's condition were achieved, highlighting the importance of tailored treatment plans.

## References

[1] Morishita Y, Matsushita A, Maeda T, Ueta T, Naito M, Shiba K (2015). Rapid progressive clinical deterioration of cervical spondylotic myelopathy. Spinal Cord.

[2] Kumar K, Taylor RS, Jacques L (2007). Spinal cord stimulation versus conventional medical management for neuropathic pain: a multicentre randomised controlled trial in patients with failed back surgery syndrome. Pain.

[3] Slangen R, Schaper NC, Faber CG (2014). Spinal cord stimulation and pain relief in painful diabetic peripheral neuropathy: a prospective two-center randomized controlled trial. Diabetes Care.

[4] Taylor RS, De Vries J, Buchser E, Dejongste MJ (2009). Spinal cord stimulation in the treatment of refractory angina: systematic review and meta-analysis of randomised controlled trials. BMC Cardiovasc Disord.

[5] Hoikkanen T, Nissen M, Ikäheimo TM, Jyrkkänen HK, Huttunen J, von Und Zu Fraunberg M (2021). Long-term outcome of spinal cord stimulation in complex regional pain syndrome. Neurosurgery.

[6] Kapural L, Deer T, Yakovlev A (2010). Technical aspects of spinal cord stimulation for managing chronic visceral abdominal pain: the results from the national survey. Pain Med.

[7] Mekhail NA, Mathews M, Nageeb F, Guirguis M, Mekhail MN, Cheng J (2011). Retrospective review of 707 cases of spinal cord stimulation: indications and complications. Pain Pract.

[8] Manfield J, Bartlett R, Park N (2019). Safety and utility of spinal magnetic resonance imaging in patients with high-frequency spinal cord stimulators: a prospective single-centre study. Stereotact Funct Neurosurg.

[9] Rubino S, Adepoju A, Kumar V, Prusik J, Murphy N, Owusu-Sarpong S, Pilitsis JG (2016). MRI conditionality in patients with spinal cord stimulation devices. Stereotact Funct Neurosurg.

[10] Akter F, Kotter M (2018). Pathobiology of degenerative cervical myelopathy. Neurosurg Clin N Am.

[11] Nouri A, Tetreault L, Singh A, Karadimas SK, Fehlings MG (2015). Degenerative cervical myelopathy: epidemiology, genetics, and pathogenesis. Spine (Phila Pa 1976).

[12] Fehlings MG, Skaf G (1998). A review of the pathophysiology of cervical spondylotic myelopathy with insights for potential novel mechanisms drawn from traumatic spinal cord injury. Spine (Phila Pa 1976).

[13] Kumar K, Wilson JR, Taylor RS, Gupta S (2006). Complications of spinal cord stimulation, suggestions to improve outcome, and financial impact. J Neurosurg Spine.

[14] Wloch A, Capelle HH, Saryyeva A, Krauss JK (2013). Cervical myelopathy due to an epidural cervical mass after chronic cervical spinal cord stimulation. Stereotact Funct Neurosurg.

[15] Kim GU, Chang MC, Kim TU, Lee GW (2020). Diagnostic modality in spine disease: a review. Asian Spine J.

[16] Alimi M, Njoku I, Hofstetter CP (2016). Anterior cervical discectomy and fusion (ACDF): comparison between zero profile implants and anterior cervical plate and spacer. Cureus.

[17] Reynolds AF, Shetter AG (1983). Scarring around cervical epidural stimulating electrode. Neurosurgery.

